# How do rest-pause and sarcoplasma stimulating training models affect metabolic and psychoaffective responses in bodybuilding athletes compared to traditional training?

**DOI:** 10.3389/fspor.2024.1467762

**Published:** 2024-10-29

**Authors:** Gustavo P. L. Almeida, Gustavo A. João, Mário A. Charro, Wilian de Jesus Santana, Carlos Eduardo Rosa da Silva, Danilo S. Bocalini, Érico C. Caperuto, Aylton J. Figueira

**Affiliations:** ^1^ Translational Physiology Laboratory, São Judas Tadeu University, São Paulo, Brazil; ^2^ Department of Physical Education Laboratory, Metropolitanas Unidas College, São Paulo, Brazil; ^3^ Department of Physical Education Laboratory, Federal University of Espírito Santo, Vitória, Brazil

**Keywords:** strength training, rest interval, acute effect, lactate, rest pause

## Abstract

**Introduction:**

Strength training (ST) is a strategy to enhance quality of life through increased strength, muscle hypertrophy, and functional capacity. Training systems are associated with manipulation of volume and intensity, generating different stimuli, such as Rest-Pause (RP) and Sarcoplasmic Stimulating Training (SST). These systems induce greater mechanical and physiological stress, leading to increased strength and muscle hypertrophy. However, the metabolic and psycho-affective effects of advanced systems in experienced practitioners remain inconclusive. The purpose of the study is to analyze the acute effects of RP, SST, and Traditional (TMS) systems on metabolic and psycho-affective responses in adult men.

**Methods:**

This experimental crossover study assessed 15 subjects (30.38 ± 2.06 years; 88.40 ± 6.50 kg; 1.74 ± 0.07 cm) experienced in ST, evaluated under TMS, RP, and SST during flat bench press and leg press 45° exercises. Body composition, muscular strength via 1-RM testing, lactate concentration (LAC), and psycho-affective measures (Rating of Perceived Exertion-RPE; Visual Analog Scale-VAS; Feeling Scale-FS) were determined. Statistical analysis was performed using the Minitab software, with *p* ≤ 0.05, IC-95%).

**Results:**

The finals results showed SST exhibited a 38.10% lower LAC concentration post-training session compared to TMS, while RP showed 37.20% lower LAC concentration than TMS post-session. Average RPE values for RP and SST were higher (8.50 ± 1.10 and 8.60 ± 0.90, respectively) than TMS (6.00 ± 1.10). VAS displayed higher average values for RP and SST (8.00 ± 2.00 and 8.00 ± 1.00, respectively) compared to TMS (5.00 ± 1.00), with affective ratings indicating positive values for TMS and values between 0 and −5 for RP (40%) and SST (60%) post-training sessions, suggesting that RP and SST induced less affective response than TMS.

**Discussion:**

The results lead to the conclusion that manipulation of training volume and intensity led to higher RPE and pain (VAS). The data suggest that inappropriate prescription of these systems could lead to greater displeasure, leading us to hypothesize that a higher likelihood of discontinuation from strength training programs would occur, suggesting that greater repetition volumes (RP and SST) should be targeted at individuals with a higher training level.

## 1 Introduction

Strength training (ST) stands as a pivotal intervention for enhancing overall health parameters (1). Its efficacy extends to improving athletic performance and facilitating activities of daily living by fostering gains in both strength and muscle mass (2). Key adaptations observed in ST include heightened strength levels and muscle hypertrophy, driven by the orchestrated release of myokines (2–5). Notably, the methodological framework governing training practices, encompassing parameters such as intensity (load magnitude in kilograms, execution velocity, recovery intervals, range of motion), and volume (repetition and set count, exercise sequencing, and weekly training frequency), significantly influences these physiological responses (4, 6–8). The manipulation of training prescription variables elicits diverse adaptive outcomes, thus addressing potential plateaus in physiological progression (2). Training systems as Rest-Pause (RP) and Sarcoplasmic Stimulating Training (SST) offer avenues for regulating intensity during ST sessions. The RP system considers small intra-set pauses (10–20 s) after concentric failure (9). Short intervals allow the maintenance of high loads and an increase in the total training session volume. On the other hand, the SST is divided into two steps, with different intensities: the first step (70%–80%—1-RM), with sets performed until concentric failure and 20-s intervals between sets, and it is finished until exhaustion for a single repetition. In the second step, the intensity is reduced by 20%, following the same procedure as the first step. SST are based on increase total RT session volume (10, 11).

High RT volume (sets per week and repetitions per exercise) is associated with greater gains in hypertrophy compared to lower training volume in both trained and untrained subjects (12). On the other hand, higher intensity and training volume are associated with lower pleasure/enjoyment, as a psychosocial indicator, in the training session, regardless of the adopted periodization model (13). Thus, the aim of the present study was to analyze the acute effect of three strength training systems (RP, SST and Traditional Multiple Sets-TMS) on the metabolic and psychophysiological response and the association with Total Load in trained adult men.

## 2 Materials and methods

### 2.1 Participants

Fifteen resistance-trained men participated in this study. The average ± SD (range) age, height, and body mass of the participants were 30.38 ± 2.06 (25–35) years, 174.9 ± 0.07 (157–179) cm, and 88.40 ± 6.50 (78.9–100.1) kg, respectively. All participants had 3 years of resistance training experience, with a frequency of at least 5 training days per week, maintaining an average of 22–26 weekly sets for each muscle group. Additionally, they were required to have experience performing the bench press and 45° leg press. The subjects were free from any musculoskeletal disorders and had no history of injury (pain, discomfort) in the trunk, upper, or lower limbs in the past three months. All recruited subjects were bodybuilders with prior knowledge and experience in performing various training systems in their sessions, enabling them to execute the three different strength training protocols studied: Traditional Multiple Sets (TMS), SST (SST), and Rest-Pause (RP). In the familiarization session, participants performed 15 repetitions of each exercise at 50% of their 1-RM to ensure proper lifting technique. The subjects abstained from their regular training for 3–7 days before the testing sessions. Participants received prior clarification regarding their participation in the study, as described in the informed consent form (ICF) based on CNS Resolution No. 196/96, in accordance with the ethical principles standardized by CNS Resolution No. 466/2012, following the Helsinki Declaration.

### 2.2 Experimental design

The experimental and comparative crossover research in which subjects were randomized to perform three training systems and the study was conducted in three phases: (Phase 1)—weeks 1, 2, and 3: anthropometric variables were measured and maximum strength values were determined; familiarization with exercises and training systems and perception scales of effort and affectivity; (Phase 2)—week 4–10: experiments with exercise sessions. After the strength training sessions, subjective perception of effort, affectivity, and perception of pain/discomfort were collected. Lactate concentrations were assessed pre and post training protocols. Daily dietary habits and water consumption were instructed to follow the strategies established prior to the present study.

### 2.3 Anthropometric evaluation

The anthropometric evaluation determined the body mass (BM) and body composition was determined using the InBody® model H20B. Height was measured with a stadiometer with precision in millimeters. Circumferences of the chest, waist, abdomen, hips, right and left biceps, right and left forearm, right and left thigh, and right and left leg were determined (Table 1). Body composition assessment was initially conducted using bioimpedance, however, it was excluded after the pilot study due to the lack of data reliability across consecutive evaluation days. The bodybuilding cohort adhered to specific hydration and supplementation protocols, which introduced variability in both inter- and intragroup results in bioimpedance data. As all evaluations were performed in the morning, and the inconsistency of the bioimpedance data, anthropometric measurements and circumference assessments were adopted as more reliable protocols.

**Table 1:** Sample characteristics, anthropometry of trained subjects to three training models.

	Mean	SD
Age (years)	30.38	2.06
Body mass (Kg)	88.40	6.50
Height (m)	1.74	0.07
BMI (kg/m^2^)	29.20	2.20
Body Fat (%)	13.00	4.20
Lean body weight (Kg)	67.37	8.07
Fat body weight (Kg)	21.05	4.84
Chest CIRC (cm)	105.33	8.28
Waist CIRC (cm)	82.57	6.51
Abdominal CIRC (cm)	84.60	5.60
HIP CIRC (cm)	97.38	5.88
Right biceps CIRC (cm)	38.47	3.66
Left biceps CIRC (cm)	38.20	4.09
Right forearm CIRC (cm)	32.20	4.37
Left forearm CIRC (cm)	32.40	4.53
Right thigh CIRC (cm)	64.13	4.66
Left thigh CIRC (cm)	64.47	4.80
Right leg CIRC (cm)	37.83	2.35
Left leg CIRC (cm)	37.80	2.31

CIRC, circunference.

### 2.4 1-repetition maximum test (1-RM)

Upper and lower body maximum strength was assessed by one-repetition maximum (1-RM) test, following the ACSM (5) protocol. All volunteers were informed about the testing routine prior to its execution. The 1-RM test and retesting sessions were conducted on different days, with a 72-h interval between tests. The exercises tested included bench press and the 45° leg press. The protocol consisted of 5 min of low-intensity walking on a treadmill, followed by a specific warm-up allowing 1 set, 20 repetitions, with participant self-selected load. The 1-RM test protocol started after a 3-min rest load. Up to six attempts were permitted to identify the maximum weight the volunteer could lift in one repetition, with a 5-min rest interval between attempts. The first attempt used submaximal loads, with an increase of 10% kg for the 45° leg press and 5% kg for the bench press for subsequent attempt, gradually approaching the 1-RM. The maximum load was defined as the last weight at which the individual performed the movement with appropriate technique, execution, and range of motion, according to Brown and Weir (14). If the maximum load was not identified within six attempts, a new test was conducted 48 h after the previous test.

### 2.5 Blood lactate analyses

Were performed by measuring lactate concentrations (LAC) at rest, pre and post training sessions were performed on the fingertip of the dominant hand (15) using the portable monitor brand Roche® Model AccutrendPlus and Accusport BM-Lactate® reagent strips.

### 2.6 Rationing perception exertion

Three instruments were applied at the end of the training sessions: (1) Borg's Rating of Perceived Exertion (RPE) CR-10 Scale (which determines perceived exertion during training sessions); (2) Visual Analog Scale (VAS), which associates pain and discomfort with training; (3) Feeling Scale (FS), which determines the pleasure/displeasure relationship on an 11-point scale (+5, 0, −5).

### 2.7 Experimental sessions

**Figure 1:**
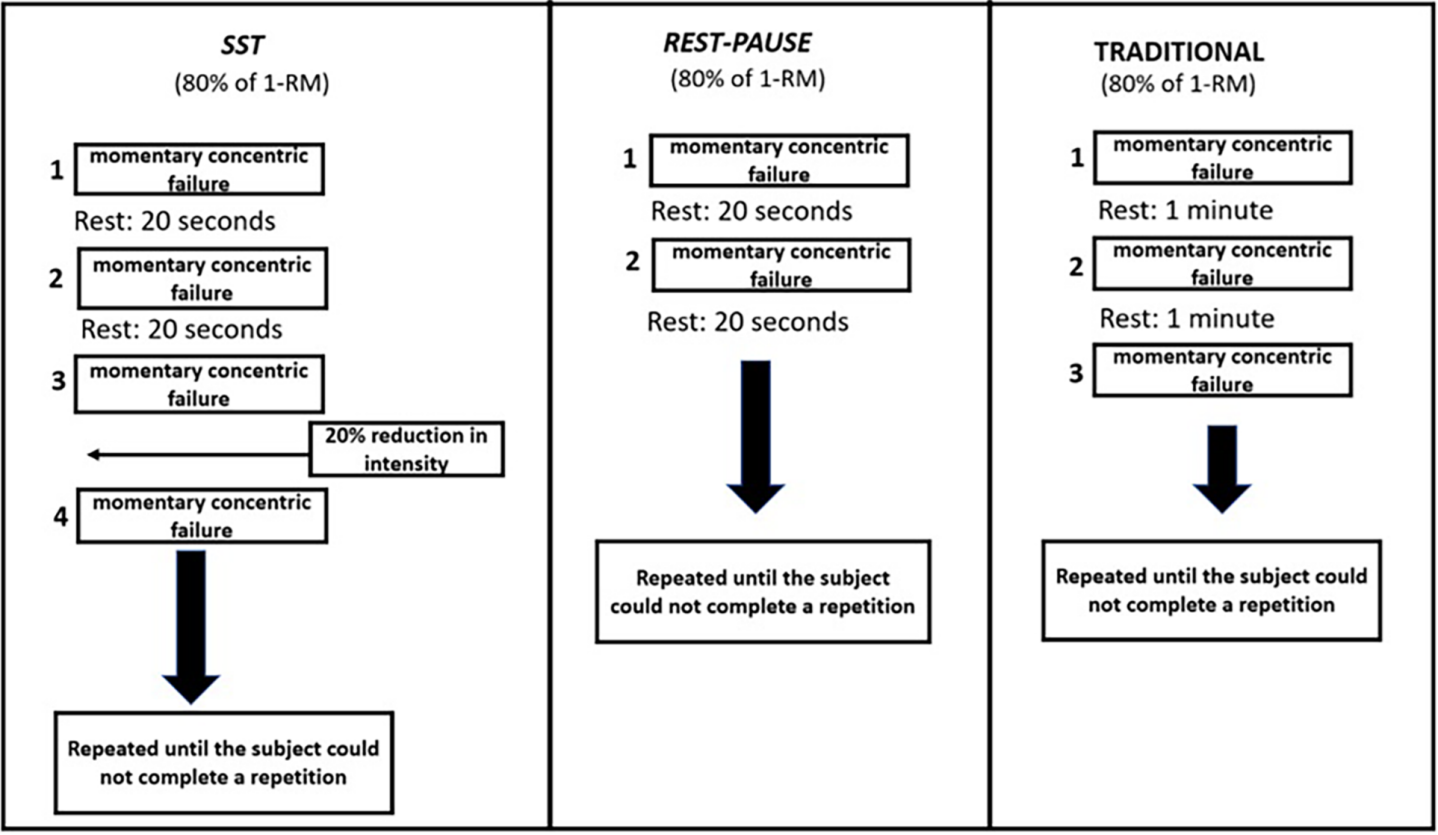
Training protocols design applied in bodybuilders athletes.

The study was designed in three non-consecutive days training sessions. The RP, SST, and TMS systems were randomized within the bench press and leg press 45°. MST was consistently performed last, as the SST and RP involve repetitions and sets that vary among individuals. The SST and RP methods contribute to high training volumes with reduced rest intervals between sets. Consequently, the equalization of total training volume (sets × repetitions per exercise) was achieved by averaging the movements between RP and SST, forming the basis for the TMS protocol. Following the volume equalization, the total training load (TL) was calculated for each subject and exercise using the following formula: TL = sets × repetitions × load (intensity). Warm-up before the sessions consisted of three sets of 15 repetitions on bench press and leg press 45°, at 50% of 1-RM and with 2 min of rest between sets. The protocols were equalized by total volume between the systems. The protocols used were: (1) SST, performed in two passes. In the first pass, subjects performed sets until momentary concentric failure, followed by a 20-s rest interval after each failure. When the subject could not perform a single maximum repetition (1-RM), the second pass began with a 20% reduction in intensity (kg). The protocol was repeated until the subject could not complete a repetition; (2) RP, where the subjects performed exercises until momentary concentric failure, followed by a 20-s rest interval between sets. The protocol was concluded when the subject could not complete at least one repetition; (3) TMS, where participants performed multiple sets equalized to SST and RP until momentary concentric failure, with a predefined 1-min recovery between sets. The training protocols follows (Figure 1).

### 2.8 Statistical analyses

The analysis utilized descriptive statistics (mean and standard deviation, both descriptive and inferential) conducted with the statistical software Minitab, employing a significance criterion of 95%. Normality was assessed using the Shapiro–Wilk test for all variables. One-Way ANOVA (*α* = 0.05), followed by Tukey's *post hoc* test, was employed to compare the TMS-SST-RP outcomes. The sample size required was estimated using G*Power 3.1 software (version 3.1.9.4) (16), with effect size [ES] = 0.5, from the previous study comparing systems strength programs (11). *A priori* power analysis assuming an estimating error of *α* = 0.05, and *β* = 0.99, suggested a sample size of 9 participants to achieve a statistically significant difference between the strength training systems. Thus, the use of *n* = 15 enabled a statistical power = 1.00. Pearson's linear correlation was utilized to explore the relationship between the Total Load of each session and RPE; Total Load of each session and VAS; Total Load of each session and FS. Also, Total Load of each session was correlated to LAC concentration at the end of training sessions. Additionally, correlations were examined between the intensities (in kg) of leg press 45° and bench press exercises and psycho-affective responses. The magnitude of correlations, correlation values were classified as follows (17): (0.0; 0.1), trivial; (0.1; 0.3), small; (0.3; 0.5), moderate; (0.5; 0.7), large; (0.7,0.9), very large; and (0.9; 1.0), nearly perfect. The Tukey HSD *post hoc* test and the Cohen's *d* tested the significances and the effect size of differences between factors. The following magnitude inferences were made for the Cohen's *d*: 0.0–0.2, trivial effect; 0.2–0.6, small effect; 0.6–1.2, moderate effect; and 1.2–2.0, large effect. Statistical procedures were performed for 95% significance.

## 3 Results

Relative Strength—Analyzing the outcomes pertaining to relative strength (Mean relative strength = weight lifted [kg]/body mass [kg]—Heyward's formula, 1997), the relative strength (bench press) was classified (0.90 ± 0.27; good), and leg press 45° was deemed “superior” (3.63 ± 1.16). These findings denote the sample's as trained subjects, thereby fulfilling the study's inclusion criteria.

Load and Volume—The assessment of the load lifted (kg) during bench press (79.00 ± 23.30 kg; *p* = 0.086) and leg press 45° (255.40 ± 79.20 kg; *p* = 0.106) demonstrated comparable outcomes between RP and TMS. SST exhibited lower intensity during both bench press (63.00 ± 18.70 kg; *p* = 0.086) and leg press 45° (204.30 ± 63.30 kg; *p* = 0.106). Notably, SST with two trials, and 20% reduced intensity (kg), impacting exercise load determination. Regarding bench press load outcomes, SST and RP presented higher loads (11,197 ± 4,039 kg and 5,072 ± 2,166 kg; *p* < 0.05) compared to TMS (7,088 ± 1,664 kg; *p* < 0.05), suggesting that both SST and RP induce greater training loads than TMS. Similarly, leg press 45° results at SST and RP exhibited higher training loads (37,091 ± 14,530 kg and 21,485 ± 9,500 kg; *p* < 0.05) than TMS (16,436 ± 5,609 kg; *p* < 0.05). Regarding total training volume analysis, the outcomes in Table 2 revealed that SST and RP demonstrated higher loads (48,288 ± 18,300 kg and 26,557 ± 11,208 kg; *p* < 0.05) than TMS (23,524 ± 6,818 kg; *p* < 0.05) (Table 2).

**Table 2:** Total load of the training session in the three systems.

Total load									
System	*N*	Mean	SD	IC de 95%	Δ%	Effect size	*D* Cohen	Classification EF	*p* value
TMS	15	23.52	6.82	(16.750; 30.298)					0
SST	15	48.29	18.30	(41.514; 55.062)	105.27%	0.66	1.80	Moderate	
Rest pause	15	26.56	11.21	(19.783; 33.331)	12.89%	0.16	0.32	Ignored	

Lactate concentration follows in Table 3, showing that TMS had the highest concentration (10.45 ± 2.44 mmol/L; *p* < 0.05) compared to SST (7.13 ± 1.67 mmol/L; *p* < 0.05) and RP (8.13 ± 2.17 mmol/L; *p* < 0.05).

**Table 3:** Lactate concentration pre- and post-training sessions.

Lactate (mmol/L)											
System	*N*	M. pré	SD	M. post	SD	IC de 95%	Δ%	Effect size	*D* cohen	Classification EF	*p* value
TMS	15	1.63	0.38	10.45	2.44	(7.690; 9.950)					0
SST	15	1.67	0.25	7.13	1.67	(4.330; 6.590)	−38.1%	0.61	1.56	Moderate	
REST PAUSE	15	1.70	0.36	8.13	2.17	(5.297; 7.557)	−37.2%	0.45	1.00	Small	

M. pre, mean pre-training session; M. post, mean post-training session.

Psychosocial response (RPE) in RP and SST (8.50 ± 1.10 and 8.60 ± 0.90; *p* < 0.05 respectively) were higher than in TMS (6.00 ± 1.10; *p* < 0.05), suggesting that the relationship between volume and intensity had a greater effect in denser training sessions (Table 4).

**Table 4:** Post-training session values of rate of perceived exertion in the three systems.

Effort (0–10 Borg Scale)									
System	N	Mean	SD	IC de 95%	Δ%	Effect size	*D* Cohen	Classification EF	*p* value
TMS	15	6.00	1.10	(5.459; 6.541)					0
SST	15	8.60	0.90	(8.059; 9.141)	43.33%	0.79	2.58	Moderate	
REST PAUSE	15	8.50	1.10	(7.992; 9.075)	42.17%	0.75	2.27	Moderate	

Pain and discomfort perception (VAS) in RP and SST (8.00 ± 2.00 and 8.00 ± 1.00; *p* < 0.05 respectively) were higher than in TMS (5.00 ± 1.00; *p* < 0.05), suggesting that systems with higher volumes promoted a greater sensation of pain and discomfort (Table 5).

**Table 5:** Post-training session values of pain perception in the three systems.

Pain and discomfort (0–10 points—AVS)									
System	*N*	Mean	SD	IC de 95%	Δ%	Effect size	*D* Cohen	Classification EF	*p* value
TMS	15	5	1	(4.591; 5.839)					0
SST	15	8	1	(7.428; 8.705)	51.29%	0.82	2.88	High	
REST PAUSE	15	8	2	(7.228; 8.505)	55.13%	0.71	2.02	Moderate	

Affection, associated with pleasure/displeasure (FS), in TMS predominantly resulted in positive sensations, while in RP and SST, there was a greater tendency for negative sensations (Table 6).

**Table 6:** Post-training session values of affectivity in the three systems.

Affectivity (11 Points—FS)									
System	*N*	Mean	SD	IC de 95%	Δ%	Effect Size	*D* Cohen	Classification EF	*p* value
TMS	15	2.20	1.57	(0.643; 3.757)					0.076
SST	15	−0.27	3.22	(−1.824; 1.290)	112.00%	0.36	0.78	Small	
REST PAUSE	15	1.53	3.74	(−0.024; 3.090)	−30.00%	0.00	0.00	Ignored	

## 4 Discussion

The present study analyzed the acute effects of metabolic, psycho-affective, and psychophysiological responses in adult subjects with experience in ST, undergoing training in the RP, SST, and TMS systems. Achieving a higher training volume in a shorter training duration is a strategy to increase muscle hypertrophy (18). Haun et al. (19) conducted a study involving 31 trained male participants (21.5 ± 2.3 years; 83.4 ± 10.4 kg; 178.2 ± 6.4 cm) over a six-week, revealing that high training volume is conducive to hypertrophy, particularly through sarcoplasmic hypertrophy. A recent systematic review by Krzysztofik et al. (10), encompassing 38 articles, concluded that higher training volume is positively correlated with increases in muscle mass. The authors emphasized that manipulating training volume (the total number of sets and repetitions) is essential for optimizing hypertrophic outcomes. Further corroborating this assertion, Zourdos et al. (20) demonstrated that increased volume across training sessions significantly enhances hypertrophic adaptations. Prestes et al. (21) showed that SST and RP contribute to increasing training volume, being more appropriate for hypertrophy in trained male. On the other hand, high resistance training volume contribute to lactate accumulation (22), considered a key factor for hypertrophic responses, due to anabolic signaling pathways, such as mTORC1. Our data showed that TMS promoted higher LAC concentration (8.82 ± 2.50 mmol/L) than SST (5.46 ± 1.71 mmol/L) and RP (6.43 ± 2.23 mmol/L), *p* < 0.05 difference between TMS-SST (3.36 mmol/L) and TMS-RP (2.38 mmol/L). Similar trend was found to Almeida et al. (11), that analyzed two variations of SST and TMS with no significant differences (pre-post) LAC concentrations. Martins-Costa et al. (23) observed that higher total volume the protocol and slower contraction movement generated higher LAC than lower total volume protocol.

Our results LAC concentrations presented higher in the TMS system due to residual accumulation, given that this system was performed last in the training sequence. Our hypothesis is that since the RP and SST systems exhibited higher intensities, there was a tendency for a progressive residual accumulation of LAC between evaluation days (SST = 7.13 ± 1.67 mmol/L; RP = 8.13 ± 2.17 mmol/L; TMS = 10.45 ± 2.44 mmol/L). Therefore, it is important to highlight that evaluating LAC with residual values between days may interfere with the analysis of training effects.

This residual accumulation could affect performance in strength training. Nóbrega et al. (24), presented that LAC increase after intense strength training sessions can impair muscle recovery, decrease strength and power in subsequent sessions with high LAC. Similarly, Jenkins et al. (25) asserts that after an intense training session, LAC remains elevated after training due to training volume, which can negatively affect subsequent training performance.

The resistance training week frequency and high-intensity training programs may contribute to greater residual LAC accumulation, as SST and RP. Individuals who train with high frequency tend to show higher levels of LAC at rest after session, which may require specific recovery strategies to lactate removal (18).

Our findings revealed a significantly higher Rate of Perceived Exertion (RPE) in the Resistance Training (RP) and Single-Set Training (SST) conditions (8.50 ± 1.10 and 8.60 ± 0.90; *p* < 0.05, respectively) compared to the Traditional Multi-Set (TMS) condition (6.00 ± 1.10; *p* < 0.05). The effort expended to reach concentric failure is a variable that contributes to increased perception of exertion and is associated with greater total training volume and reduced rest intervals (RI) (18). Recent literature indicates that sets performed with repetitions approaching failure are correlated with heightened perceived exertion and neuromuscular fatigue (26, 27). Furthermore, the rest interval between sets plays a critical role in energy depletion and the clearance of metabolites in active muscle tissue. Shorter recovery intervals between sets have been shown to exacerbate fatigue levels (28). Additionally, reduced RIs significantly amplify muscle soreness and subjective ratings of discomfort (29), both of which are associated with increased unpleasantness.

The average VAS values in RP and SST (8.00 ± 2.00 and 8.00 ± 1.00; *p* < 0.05 respectively) were higher than TMS (5.00 ± 1.00; *p* < 0.05). Our results indicate that higher intensity protocols induce a reduction in K+ levels in muscle cells, affecting cell excitability and pain sensation, reduced RI levels increase muscle, blood acidity, and acid-base buffering, increasing pain and discomfort sensation (30).

Our results of affectivity in RP and SST align with Ribeiro et al. (31), where higher intensities led to a greater displeasure sensation. In another study, LAC accumulation led to greater pain and discomfort sensation, resulting in a higher displeasure sensation during exercise (32). These results are inconsistent with those observed in the current study, where higher lactate (LAC) levels were recorded in the TMS condition. However, pleasure and displeasure ratings remained positive despite elevated LAC concentrations. We hypothesize that the experience and understanding of exercise execution do not significantly influence the perception of pleasure, contrary to the expectations typically associated with novice individuals. This hypothesis is supported by findings from Stultz et al. (33), which indicated that experienced athletes exhibit a distinct pain tolerance and pleasure response that differ from those of novices, suggesting a complex interplay between training history and subjective exercise experiences.

In the correlation analysis between Total Load and Rate of Perceived Exertion (RPE), presented weak correlation in RP (0.09). The SST, followed similar trend (−0.16), as well TMS (−0.21). This suggests that an increase in Total Load is associated with a slight reduction in RPE.

The correlation between Total Load and muscle discomfort revealed a negligible correlation to RP (0.00), suggesting that total training load is not related to muscle soreness. Similarly, the SST condition (0.38) indicated a slight association with Total Load. The TMS presented a weak correlation (0.14) with Total Load and muscle discomfort. These data suggest that experienced subjects exhibit a reduced perception of discomfort in systems with higher volume and intensity.

The affectivity was assessed in relation to Total Load, revealing a very low correlation in the RP condition (−0.09), implying that total training load does not significantly affect affectivity. In the SST condition, a weak correlation of −0.17 was observed, indicating no meaningful association between Total Load and affectivity. In the TMS condition, a moderate negative correlation of −0.51 was identified, suggesting that an increase in total training load is associated with a decrease in affectivity among trained subjects (Table 7).

**Table 7:** Correlation between the total load of each system in the study with psycho-affective responses and lactate concentration at the end of training sessions.

Total load correlation			
	Rest-pause	SST	Traditional
Load × Perception of Effort	0.09	−0.16	−0.21
Load × Discomfort	0.00	0.38	0.14
Load × Affectivity	−0.09	−0.17	−0.51
Load × Lactato	0.51	0.34	−0.27

These findings are supported by recent studies that indicate RPE often demonstrates low correlation values across different training modalities, suggesting that perceived effort does not tightly align with training metrics (34). Additionally, discomfort and affectivity appear to interact with training load in nuanced ways, highlighting the psychological components of resistance training (35).

Considering the association between Total Load and LAC, we observed moderate correlation (0.51) in RP. Thus, higher total load is associated with a significant increase in lactate production during resistance training, indicating that the manipulation of volume and intensity is crucial for maximizing metabolic responses (36, 37). In the SST system, the correlation was weak (0.34), as well as in TMS. The intensity of leg press 45° presented a weak correlation to perceived exertion (−0.22) in RP, similar to SST (−0.17). On the other hand, the association of RPE (leg press 45°) showed a moderate correlation with intensity (−0.54), suggesting the importance of perceived effort as training intensity control (38) (Table 7).

In the current investigation of the correlation between 45° leg press intensity and subjective discomfort perception, a negligible correlation coefficient was observed for RP (*r* = 0.00), with weak positive correlations for SST (*r* = 0.38) and TMS (*r* = 0.14). These data suggest that, despite enhanced adherence and progressive neuromuscular adaptations, the interplay between mechanical load and nociceptive feedback remains relatively invariant across heterogeneous resistance training paradigms. Smith and Jones (39) postulate that training tenure and the psychophysiological milieu during exertion are critical moderators of the subjective experience of discomfort and perceived exertion under high-intensity loading conditions. The authors emphasize the integral role of psychobiological factors, particularly attentional focus, pain tolerance, and intrinsic motivation, in mediating the dissociation between objective physical strain and subjective discomfort, further hypothesizing that athletes with advanced training experience exhibit enhanced capacity for central fatigue resistance and pain modulation through adaptive neural mechanisms.

A consistent pattern was noted in the correlation between 45° leg press intensity and affective valence, with RP (*r* = 0.00), SST (*r* = −0.17), and TMS (*r* = −0.51) showing weak negative correlations. These findings corroborate existing evidence suggesting that exercise intensity exerts differential effects on affective responses, particularly in strength-oriented protocols as opposed to endurance-based regimens. Williams and Brown (40) propose that such variability in affective responses may be mediated by the interplay between autonomic regulation, central fatigue thresholds, and the capacity for psychophysiological stress management. Furthermore, higher intensities, especially in protocols with significant external loading, are associated with pronounced neuroendocrine responses that may exacerbate the affective burden, as resistance training places considerable demand on both cognitive and affective domains (40) (Table 8).

**Table 8:** Correlation between the intensities (kg) of the leg press and bench press in each system of the study with psycho-affective responses and lactate concentration at the end of the sessions.

Correlation of intensities						
Correlation	Rest-pause	LEG press		Rest-pause	Bench press	
		SST	Traditional		SST	Traditional
Intensity × Perception of Effort	−0.22	−0.17	−0.54	−0.05	−0.19	0.22
Intensity × Discomfort	0.00	0.38	0.14	0.00	0.38	0.14
Intensity × Affectivity	0.00	−0.17	−0.51	−0.09	−0.17	−0.51
Intensity × Lactate	0.51	0.34	−0.27	0.51	0.34	−0.27

In contrast, during the bench press exercise, the correlations between intensity and perceived exertion were RP (*r* = −0.05), SST (*r* = −0.19), and TMS (*r* = 0.22), indicating weak and inconsistent relationships across different scales of perceived exertion. Additionally, the correlation between bench press intensity and discomfort perception followed a similar pattern: RP (*r* = 0.00), with weak positive correlations for SST (*r* = 0.38) and TMS (*r* = 0.14). Lea et al. (41) conducted an in-depth meta-analysis on perceived exertion, identifying exercise duration, prior training experience, and environmental conditions as key determinants that modulate the subjective interpretation of effort. Their findings suggest that familiarity with a given exercise can reduce the perception of discomfort, thus enhancing task focus and goal-oriented performance in resistance training contexts.

Notably, the association between bench press intensity and affective responses exhibited a negative correlation: RP (*r* = −0.09), SST (*r* = −0.17), and TMS (*r* = −0.51), the latter reflecting a moderate negative correlation. These results suggest that increasing training intensity is inversely related to affectivity, particularly in advanced resistance training protocols where the focus is on maximal strength. This observation is further supported by Garcia and Davis (42), who demonstrated that the escalation of external load contributes to the accumulation of both central and peripheral fatigue, thereby attenuating intrinsic motivation and reducing hedonic value in high-intensity strength training. The authors emphasize that the intensity-affect relationship is contingent upon the athlete's emotional and cognitive states, wherein heightened loads may be perceived either as a constructive stimulus that promotes adaptive stress responses or as a psychologically aversive stimulus that impairs performance, particularly in the presence of high training volume and cognitive strain (Table 8).

Despite the loads being equalized across the three training systems, the correlation between bench press intensity and lactate concentration (LAC) revealed the following: RP (*r* = 0.51), SST (*r* = 0.34), and TMS (*r* = −0.27). These lactate values reflect not only the execution time but also the distinct relative intensities inherent to each system. Thompson and White (43) underscore that lactate production is highly sensitive to exercise intensity, with higher concentrations observed in protocols that emphasize high-intensity efforts, thereby suggesting increased metabolic stress. Their findings indicate that elevated lactate levels serve as a biomarker for metabolic demand and training efficacy, as sustained increases in lactate are positively correlated with physiological adaptations such as enhanced glycolytic capacity, improved buffering of acidosis, and greater tolerance to high-intensity workloads, which may collectively contribute to improved athletic performance over time (Table 8).

## 5 Conclusions

Our results demonstrated that the manipulation of training variables led to different responses in physiological parameters, such as LAC concentration, and in psycho-affective responses. The values found in relation to the 1-RM tests align with our initial hypotheses, as the leg press 45° showed higher 1-RM and relative intensity than the bench press in both the RP and SST systems, as well as in work percentage (TMS).

The psychophysiological assessments were consistent with our hypotheses, demonstrating that these variables had greater alterations in the RP and SST protocols compared to TMS. LAC concentrations (RP and SST) were lower than in TMS, contrary to our initial hypotheses. Perceived exertion, sensations of pain and discomfort, and displeasure were higher in RP and SST, even with lower LAC concentrations compared to TMS, which was opposite to our initial hypotheses.

Additionally, the findings of the present study support the data regarding psychophysiological parameters in the context of strength training, as the variables RPE, VAS, and FS behaved as expected with the increase in training intensity and volume.

Thus, the psycho-affective responses (perceived exertion measured by RPE, pain perception measured by VAS, and affectivity measured by the Feeling Scale) presented expected scores when correlated with intensity and Total Load. These data lead us to infer that the responses of RPE and FS were likely influenced by the scores reported for pain and discomfort from the VAS, resulting in greater discomfort.

This study presented some limitations that should be considered. The sample size (*N* = 15) is small, which limits the generalization and in-depth analysis of the data, as it reduces the statistical power of the study. Additionally, since the study's sample consisted only of trained individuals with experience in strength training, this limits the applicability of the results to other populations, such as beginners, elderly individuals, or those with different fitness levels. Another important point to consider is that the study aimed to observe acute effects, meaning the results reflect only the immediate responses to the training sessions. It does not provide information on long-term adaptations or the potential chronic effects of these training systems. This is a limitation, as a chronic study over the long term could present different results and analyses. Moreover, participants were requested and reminded not to train outside of the evaluation periods and to adhere to this request to avoid interfering with the results. Overall, while the study provides important findings, these limitations should be addressed in future research to enhance the robustness and applicability of the results.

## Data Availability

The original contributions presented in the study are included in the article/Supplementary Material, further inquiries can be directed to the corresponding author.
